# *Sarcocystis*-infection of cattle in Hungary

**DOI:** 10.1186/s13071-015-0685-9

**Published:** 2015-02-04

**Authors:** Sándor Hornok, Anita Mester, Nóra Takács, Ferenc Baska, Gábor Majoros, Éva Fok, Imre Biksi, Zoltán Német, Ákos Hornyák, Szilárd Jánosi, Róbert Farkas

**Affiliations:** Department of Parasitology and Zoology, Faculty of Veterinary Science, Szent István University, Budapest, Hungary; Veterinary Clinic, Mester tanya, Bátonyterenye, Hungary; Department of Pathology, Faculty of Veterinary Science, Szent István University, Budapest, Hungary; Department and Clinic for Production Animals, Faculty of Veterinary Science, Szent István University, Üllő, Hungary; Laboratory of Virology, Veterinary Diagnostic Directorate of National Food Chain Safety Office, Budapest, Hungary; Department of Bacteriology, Veterinary Diagnostic Directorate of National Food Chain Safety Office, Budapest, Hungary

**Keywords:** Cattle, Buffalo, *Sarcocystis*, Zoonosis, Dalmeny disease

## Abstract

**Background:**

Reports on *Sarcocystis*-infection of cattle are outdated or lacking in many European countries, including those in the Central-Eastern part of the continent. Therefore, to assess the prevalence of *Sarcocystis* spp. among bovids in Hungary, a countrywide survey was initiated. In addition, fulminant deaths of four cattle, that showed clinical signs and post mortem lesions resembling acute sarcocystiosis (“Dalmeny disease”), were investigated.

**Methods:**

During the countrywide survey individual heart and oesophagus samples were collected at slaughterhouses from 151 beef cattle and from 15 buffalo, kept in 31 places of Hungary. Analysis for *Sarcocystis* spp. was carried out with conventional PCRs for the 18S *rDNA* gene and gel electrophoresis, followed by sequencing of 36 strongly positive samples. Mortality cases were evaluated by histological, molecular, bacteriological and virological analyses of samples from various organs.

**Results:**

Among slaughtered cattle the rate of *Sarcocystis*-infection was 66%. *S. cruzi* was identified as the most prevalent species in aurochs-like breed, and the zoonotic *S. hominis* in Hungarian grey cattle. Concerning the sudden deaths of cattle, *Sarcocystis*-infection could not be demonstrated in organs showing haemorrhages, but *S. cruzi* cysts were present in the muscles. In one case “*S. sinensis*” was molecularly identified in the blood (indicating sarcocystaemia). Results of analyses for bacterial/viral pathogens were negative.

**Conclusions:**

*S. cruzi* appears to be the most prevalent *Sarcocystis* sp. in cattle in Hungary, followed by the zoonotic *S. hominis*. However, the rate of infection with both species was shown to differ between cattle breeds. The suspected role of *Sarcocystis* spp. as causative agents of the fatal cases could not be confirmed.

## Background

*Sarcocystis* species are unicellular parasites that belong to cystogenic coccidia (Apicomplexa: Sarcocystidae). During their life cycle they require both an intermediate and a final host, the former usually a herbivorous and the latter a carnivorous vertebrate animal. Cattle are long-known intermediate hosts of *S. cruzi*, *S. hirsuta* and *S. hominis*, with canids, felids and humans as final hosts, respectively [1]. In addition, *S. sinensis* has been reported from cattle (recently also from Germany: [2]), but its final host remains to be elucidated and the species name was rendered *nomen nudum* [3]. Therefore “*S. sinensis*” is put between quotation marks throughout the text. Among the four bovine *Sarcocystis* spp. *S. hominis* has public health importance, causing gastrointestinal malaise [1], but “*S. sinensis*” may also elicit symptoms in humans [4] and therefore can be regarded as potentially zoonotic. Bovine sarcocystiosis was reported to entail eosinophilic myositis [5], encephalomyelitis [6] and acute death of cattle (so-called “Dalmeny-disease” caused by *S. cruzi* [7,8]).

Formerly, the identification of *Sarcocystis* spp. in the intermediate hosts required morphological analysis of cysts, but nowadays molecular biological methods (PCR and sequencing) began to overtake its diagnostic importance [9]. In this context reports on *Sarcocystis*-infection of cattle are outdated in many European countries [10]. In particular, although *Sarcocystis* spp. were reported more than a century ago in Hungary [11], and from wild ruminants and sheep during the past decades [12,13], the current significance of bovine *Sarcocystis*-infection is not known in the country, nor in the whole Central-Eastern European region [10]. Therefore the primary aim of the present study was to compensate for the lack of relevant data, by initiating a nationwide survey on *Sarcocystis*-infection of slaughtered cattle. It was also within the scope of the study to investigate four cases of fulminant deaths of cattle, reported recently in Northern Hungary, with pathologies resembling “Dalmeny disease”.

## Methods

### Molecular survey of *Sarcocystis*-infection among cattle and buffalo

**Figure 1:**
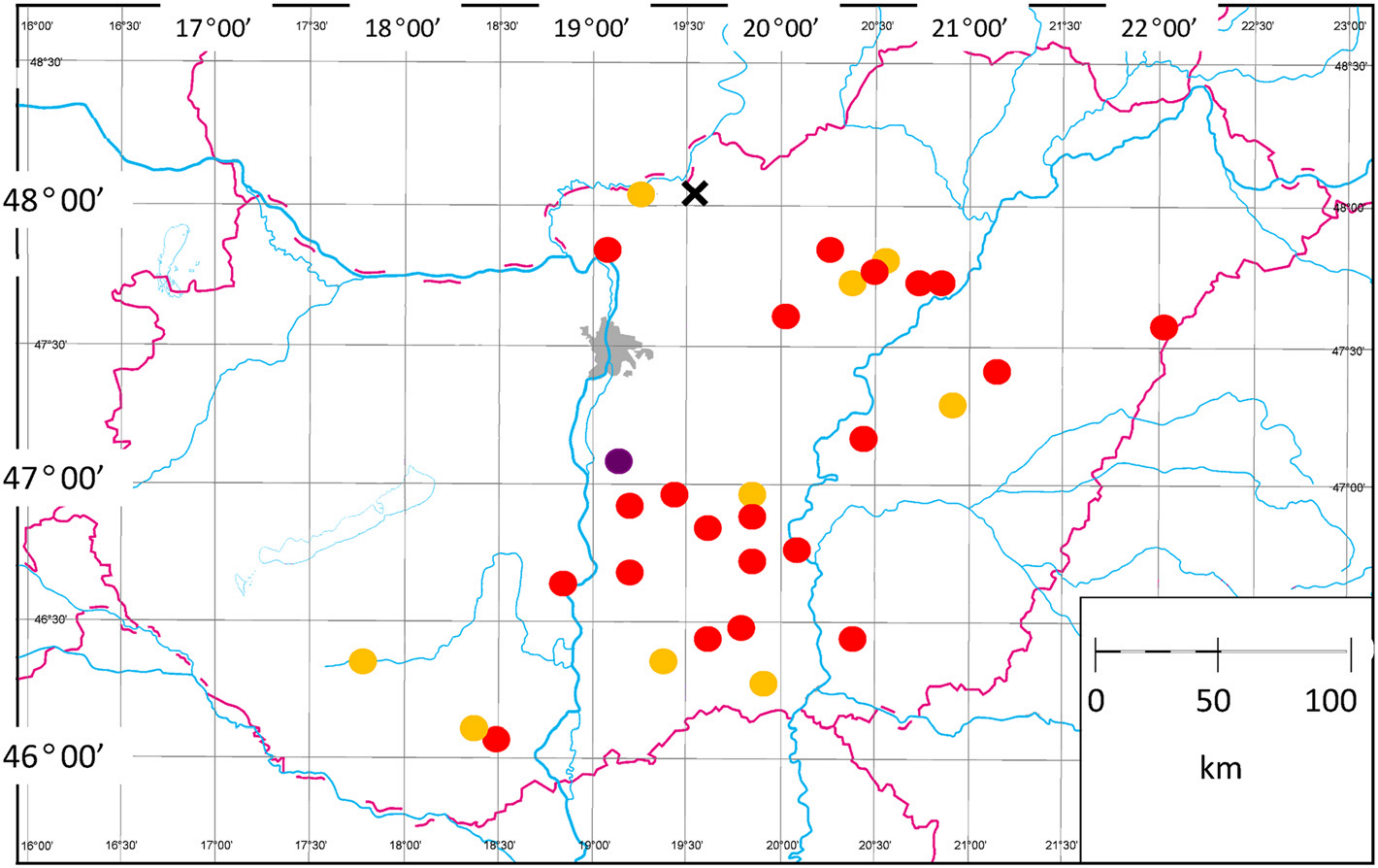
**Map of Hungary showing places of sampling, where** ***Sarcocystis*** **-infected cattle (red dots) or only uninfected ones (yellow dots) were identified.** The purple dot indicates the place where the most significant number of samples were collected (i.e. from all the buffalo and aurochs-like cattle, as well as from the majority of Hungarian grey cattle). The cross marks the place where clinical cases were noted.

Between May and August, 2014, individual samples were collected at five slaughterhouses from 151 beef cattle and 15 buffalo. Cattle had been kept in 31 places of 11 counties (Figure 1), and belonged to six breeds (Table 1). All buffalos originated from one location (Figure 1), where samples from aurochs-like and Hungarian grey cattle were also obtained. The mean age was not significantly different between buffalo and cattle or breeds of cattle (data not shown).

**Table 1:** **Results of molecular analyses according to the species/breed of tested bovids**

**Data of animals**			**Results of molecular analyses**			
			**Number (percentage) of PCR positives**	**Identified with sequencing in … samples**		
**Species**	**Breed**	**Number tested**		***S. cruzi***	***S. hominis***	**“** ***S. sinensis*** **”**
Cattle	Hungarian grey	68	49 (72%)^a^	6^a^	6	4
	Hungarian pied	56	33 (59%)^a^	10^a,b^	1	2
	aurochs-like	17	12 (71%)^a^	7^b^	0	0
	Holstein	7	4 (57%)^a^	0	0	0
	Limousin	2	1*	0	0	0
	Charolais	1	1*	0	0	0
Buffalo	-	15	0^b^	0	0	0

*Sarcocystis* spp. were identified by sequencing the product of the screening PCR (with primers COC-1, COC-2). All sequences showed 100% homology to corresponding sequences in the GenBank. Numbers in the same column with different superscript letters have statistically significant difference.

*Percentage not shown because of low sample number.

From organs that may harbour cysts of *Sarcocystis* spp., the heart and oesophageal wall was chosen, because according to several sources these are the most relevant tissues to assess the prevalence of infection [14,15]. Based on reported numbers of *Sarcocystis* cysts per gram of muscle (8–380 cysts/g: [16]), and increasing the amount of sample (i.e. 25 mg) found suitable for molecular assessment of infection in meat [17], in the present study approx. 100 mg of oesophagus and 100 mg of heart muscle per animal were processed together for molecular analysis. From these samples the DNA was extracted with the QIAamp Mini Kit (QIAGEN, Hilden, Germany) according to the manufacturer’s instructions, and including a digestion step with tissue-lysis buffer and Proteinase-K at 56°C for one hour.

### Investigation of mortality cases

The study herds consist of 385 Charolais beef cattle kept extensively in Northern Hungary (Figure 1), i.e. grazing hilly, partly forested pastures throughout the year. Herding dogs are not used. Mortality cases were reported in January, February and twice in September, 2014 (No. 1–4, respectively). After pathological examination, DNA extraction was performed with samples from the blood (in all four cases), and in cases No. 3–4 also from seven organs (the lungs, liver, kidneys, spleen, lymph nodes, heart and oesophageal wall).

For histology in cases No. 2–4, samples of the latter organs were fixed in 8% neutral buffered (in PBS, pH 7.0) formaldehyde solution for 24 hours at 4°C temperature, dehydrated in a series of ethanol and xylene, and embedded in paraffin. Eventually, 3–4 μm thick sections were cut, and routinely stained with haematoxylin and eosin.

From cases No. 3–4 all organs processed for histology were also assessed for the presence of bacterial/viral pathogens and viral cytopathogen effects (methods not shown).

### Conventional PCR for screening

All extracted DNA samples (from the muscles of 151 slaughtered cattle and 15 buffalo in the molecular survey, as well as from the blood and seven organs of the mortality cases) were screened by a method modified from Ho et al. [18]. This PCR amplifies an approx. 350 bp portion of the 18S *rDNA* gene of *Sarcocystis* spp. with the primers COC-1 (5′-AAG TAT AAG CTT TTA TAC GGC T-3′) and COC-2 (5′-CAC TGC CAC GGT AGT CCA ATA C-3′). The reaction volume was 25 μl, containing 12.5 μl QIAGEN Multiplex PCR Mastermix (2×), 9.5 μl ddH_2_O, 0.25 μl (1 μM final concentration) of each primer, and 2.5 μl template DNA. For amplification, an initial denaturation step at 94°C for 10 min was followed by 40 cycles of denaturation at 94°C for 30 s, annealing at 54°C for 30 s and extension at 72°C for 30 s. Final extension was performed at 72°C for 10 min.

Amplification was performed in a T-personal thermal cycler (Biometra, Goettingen, Germany). Purification and sequencing (Biomi Inc.: Gödöllő, Hungary) was done from samples that showed the strongest specific band of PCR product in a 1.5% agarose gel.

### Supplementary conventional PCR

This method [19] detects an approx. 500 bp long part of the 18S *rDNA* gene of several Apicomplexan genera. In the present study it was used to obtain a longer *Sarcocystis* sequence in cases No. 3–4 (from samples positive in the screening PCR), and to test blood DNA extracts for the presence of piroplasms in all four cases. The primers BJ1 (forward: 5′-GTC TTG TAA TTG GAA TGA TGG-3′) and BN2 (reverse: 5′-TAG TTT ATG GTT AGG ACT ACG-3′) were used. The reaction volume was 25 μl, i.e. 5 μl of extracted DNA was added to 20 μl of reaction mixture containing 0.5 U HotStarTaq DNA Plus polymerase (5 U/μl), 200 μM PCR nucleotid mix, 1 μM of each primer and 2.5 μl of 10× Coral Load PCR buffer (15 mM MgCl_2_ included). For amplification an initial denaturation step at 95°C for 10 min was followed by 40 cycles of denaturation at 95°C for 30 s, annealing at 54°C for 30 s and extension at 72°C for 40 s. Final extension was performed at 72°C for 5 min.

The *S. cruzi* sequence (identified from the product of this PCR) that was different from the others already reported, was submitted to the GenBank (accession number KP006498).

### Statistical analysis

Exact confidence intervals (CI) for prevalence rates were calculated at the 95% level. Mean values were compared with *t*-test, and sample prevalence data by using Fisher’s exact test. Differences were regarded significant when P < 0.05.

## Results

### Molecular survey of *Sarcocystis*-infection among cattle and buffalo

Altogether 100 cattle out of 151 (66%, CI: 58.1-73.7%) were *Sarcocystis* PCR-positive, but none of the buffalo. This means a significant difference between the two host species (P = 0.003). There was no significant difference between the rates of infection among bulls and cows (67% vs. 64%, respectively). However, the mean age of PCR-positive cattle (6.2 ± 4.4) was significantly higher, than that of PCR-negative ones (4.7 ± 4). Concerning the overall rate of PCR-positivity, no breed predisposition could be established (Table 1).

Sequencing from 36 samples revealed that, among these, *S. cruzi* was the most prevalent species (64%, CI: 46.2-79.2%), followed by *S. hominis* (19%, CI: 8.2-36%) and “*S. sinensis*” (17%, CI: 6.4-32.8%) (Table 1), with 100% sequence homology to representative sequences deposited in the GenBank. *S. hirsuta* was not identified. *S. cruzi* was significantly more prevalent in aurochs-like (7 of 7 sequenced samples), than in Hungarian grey cattle (6 of 16 sequenced samples: P = 0.007). The zoonotic *S. hominis* was identified in 38% (6 of 16) of sequenced Hungarian grey cattle samples, and only in 8% (1 of 13) of Hungarian pied samples, but this difference did not reach the level of statistical significance (P = 0.09). However, if both agents with reported public health importance (*S. hominis*, “*S. sinensis*”) are taken into account, these were significantly more frequently identified in the Hungarian grey breed, than in the aurochs-like (P = 0.007) or all other breeds together (P = 0.005) (Table 1).

### Investigation of mortality cases

In January, 2014 (case No. 1), a nine year old bull showed rapid loss of condition and was found dead one morning without a previous history of recumbency. Pathological examination revealed sunken eyes, exsiccosis, anaemia, icterus, extensive haemorrhages on the serosal surfaces, very thin (dilute) blood and similar dark contents of the urinary bladder. Molecular analysis of the blood verified sarcocystaemia caused by “*S. sinensis*”. In February, 2014 (case No. 2), a three year old cow presented nasal bleeding, and died within a few days. Post mortem lesions included generalized serous atrophy of fat, severe congestion, oedema in the lungs and haemolytic anaemia. The myocardium contained thin-walled cysts of *S. cruzi*.

In September, one week apart, fulminant deaths of two three year old cows (cases No. 3–4) were reported. Previously, both animals exhibited bleeding from the nose and vulva. The first cow was noted with dizziness and staggering during grazing, became recumbent and died abruptly in the course of five minutes. The second cow collapsed and died in one hour. Both animals were anaemic, and the latter showed signs of icterus.

**Figure 2:**
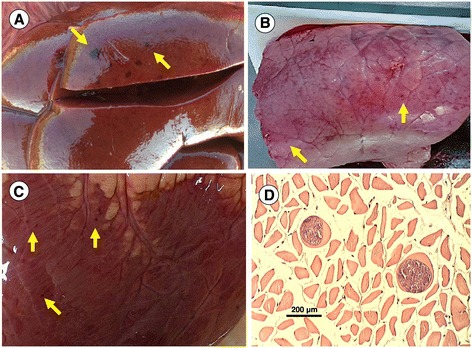
**Post mortem lesions in case No. 3: multiple haemorrhages (arrows) on the (A) kidney, (B) lungs and (C) heart.** Cysts were seen in the wall of oesophagus **(D)**.

Upon pathological examination of both animals, multiple petechial haemorrhages were seen on the surface of lungs, heart and kidneys (Figure 2A-C). The urinary bladder was full of dilute, dark reddish brown contents. The blood was very thin. Endothelial schizonts were not detected in any tissue sections. However, thin-walled cysts were found in sections of the heart and oesophagus (Figure 2D). Similarly, PCR results were negative for all evaluated organs, except for the oesophagus and myocardium. Concerning the latter, sequencing of the longer (approx. 500 bp) fragment of the 18S *rDNA* gene identified *S. cruzi*, with 99% homology (one nucleotide difference) to relevant sequences deposited in the GenBank.

In cases No. 3–4. attempts of culturing yielded negative results for aerobic, anaerobic and capnophilic bacteria, similarly to molecular analyses for IBR, BVD, bluetongue, EHD, parainfluenza-3, reo-3, corona, adeno and other bovine herpes, pox and parapox viruses. Viral cytopathogen effects were not seen in cell cultures. All samples were negative for piroplasms.

## Discussion

To the best of our knowledge, this is the first molecular investigation of the epidemiological and clinico-pathological significance of *Sarcocystis*-infection among cattle and buffalo in Central-Eastern Europe and Hungary. The 66% rate of *Sarcocystis*-infection among cattle in the present study is similar to the high prevalence (77-100%) reported in other European countries [15]. However, when comparing these data on an international level, it should be taken into account that in some molecular studies the amount of processed muscle was larger (e.g. [2]). Also similarly to other countries, most frequently *S. cruzi* was identified in cattle in Hungary. Sequences of all three *Sarcocystis* spp. obtained in the present study in various parts of Hungary were identical intraspecifically to their representative sequences in the GenBank (KC209738 for *S. cruzi*, AF006470 for *S. hominis* and KC209742 for “*S. sinensis*”).

Concerning the prevalence rates of all bovine *Sarcocystis* spp., the epidemiological situation in Hungary resembles that in Italy (with respect to *S. hominis* being the second most prevalent, and *S. hirsuta* with very low rate of positivity). On the contrary, in Germany “*S. sinensis*” was shown to be significantly more prevalent, than *S. hominis*, the latter with similar rate of infection to *S. hirsuta* [2]. Interestingly, despite the presence of the buffalo-originated agent (“*S. sinensis*”) in Hungary, verified here for the first time in the country, all locally kept Hungarian buffalo were *Sarcocystis* PCR-negative.

The zoonotic species, *S. hominis* was more frequently identified in Hungarian grey cattle, than in Hungarian pied (in 38% vs. 8% of their sequenced samples, respectively). Hungarian grey cattle (the “national breed”) are most often kept in reserves or national parks, and are frequently visited during sight-seeing. Therefore this finding may be related to more likely contact of this breed with human waste or sewage, and should draw the attention to hazards associated with unsanitary conditions of tourism (improper disposal of human excreta). On the other hand, predisposition of another cattle breed to *S. hominis* was explained by physiological factors and higher susceptibility by Timchuk *et al*. [20].

In the present study *S. cruzi* (among bovine *Sarcocystis* spp. the most pathogenic to its intermediate host) was significantly more prevalent in aurochs-like, than in Hungarian grey cattle. This implies that the former breed may be more frequently subject to the clinico-pathological effects and more severe manifestation (morbidity/mortality) of sarcocystiosis. This result may also reflect differences in the exposure to infectious sources (keeping mode, cultivation of grazed area, presence of herding dogs etc.).

Concerning sexes, some literature data support the predisposition of bulls to higher rate of *Sarcocystis*-infection [16], in part because bulls are frequently pastured close to farm buildings, thus with an increased chance for contact with final hosts [14]. However, in the present study the rate of PCR positivity was similar in bulls and cows. According to the management system in Hungarian beef farms, bulls are usually stabled when out of service. This could have thus counterbalanced the above likeliness to access infectious sources.

Higher rate of *Sarcocystis*-infection with the advance of age observed here is consistent with data reported from other countries (e.g. [14]).

In all mortality cases of the present study clinical signs that were noted are consistent with those reported by Corner et al. [7] for “Dalmeny disease”, including anaemia, icterus and haemorrhagic vaginitis. Post mortem lesions, as follows, were also indicative of acute sarcocystiosis: oedema, haemorrhagic diathesis, serous atrophy of fat [8,21] and coffee-coloured urine in the urinary bladder [7]. Experimentally, *S. cruzi* induced thrombocytopenia [22], i.e. dilute/thin blood, similarly to that observed in the present cases. Supporting the suspected clinico-pathological significance of sarcocystiosis in the study herd, sarcocystaemia caused by “*S. sinensis*” was also detected in one cattle at the time of its death.

However, the suspected etiological role of *S. cruzi* in the development of haemorrhages could not be confirmed in cases No. 3–4, because histological (and molecular) evaluation of affected organs did not reveal the presence of endothelial schizonts. Nevertheless, sarcocystiosis might still have contributed to the anaemia noted in all four cases, as the primary mechanism of red blood cell loss elicited by *S. cruzi* was reported to be haemolysis (haemorrhages *per se* were not sufficient to explain the anaemic crisis: [23]).

At the same time, no bacterial/viral pathogens were found as the cause of the above pathologies and deaths. Plant poisoning that result in coagulation disorder, most notably from consuming bracken fern [24], cannot be excluded in the background of the present fatalities, but such intoxications usually manifest as an “outbreak” [25] and frequently take a delayed course due to a cumulative effect [26], as contrasted to the isolated, fulminant cases observed in the study herd.

The *Sarcocystis* sp. in cases No. 3–4. was molecularly identified as *S. cruzi*, with one nucleotide difference in the sequenced part of its 18S *rDNA* gene from other genotypes showing the closest homology (KC209738 in [27]; or AF176934 in [28]). The 18S *rDNA* gene is regarded as highly conserved within a *Sarcocystis* sp., even over large geographical distances. Correspondingly, a uniform cattle haplotype of *S. cruzi* appears to be geographically widespread [29], and identical sequences of this species have been reported from Argentina to Japan [27]. This increases the importance of the single nucleotide divergence in the *S. cruzi* genotype of cases No. 3–4.

## Conclusions

In conclusion, *S. cruzi* appears to be the most prevalent *Sarcocystis* sp. in beef in Hungary, followed by the zoonotic *S. hominis*. The suspected role of *Sarcocystis* spp. as causative agents of the fatal cases could not be confirmed.
